# An Assessment of Siderophore Production, Mucoviscosity, and Mouse Infection Models for Defining the Virulence Spectrum of Hypervirulent Klebsiella pneumoniae

**DOI:** 10.1128/mSphere.00045-21

**Published:** 2021-03-24

**Authors:** Thomas A. Russo, Ulrike MacDonald, Sidra Hassan, Ellie Camanzo, Francois LeBreton, Brendan Corey, Patrick McGann

**Affiliations:** a Veterans Administration Western New York Healthcare System, Buffalo, New York, USA; b Department of Medicine, University at Buffalo, State University of New York, Buffalo, New York, USA; c Department of Microbiology and Immunology, University at Buffalo, State University of New York, Buffalo, New York, USA; d The Witebsky Center for Microbial Pathogenesis, University at Buffalo, State University of New York, Buffalo, New York, USA; e Multidrug-Resistant Organism Repository and Surveillance Network (MRSN), Walter Reed Army Institute of Research, Silver Spring, Maryland, USA; University of Michigan—Ann Arbor

**Keywords:** *Klebsiella pneumoniae*, hypervirulent, infection model, mice, mucoviscosity, pathogenic potential, siderophores, virulence

## Abstract

Hypervirulent Klebsiella pneumoniae (hvKp) bacteria are more virulent than classical K. pneumoniae (cKp) with resultant differences in clinical manifestations and management. It is unclear whether all hvKp isolates share a similar pathogenic potential. This report assessed the utility of siderophore production, mucoviscosity, and murine infection for defining the virulence spectrum of hvKp. Three strain cohorts were identified and defined based on the CD1 mouse subcutaneous (SQ) challenge model: (i) fully virulent hvKp strains (_fv_hvKp), lethal at a challenge inoculum (CI) of ≤10^3^ CFU; (ii) partially virulent hvKp strains (_pv_hvKp), lethal at a CI of >10^3^ to 10^7^ CFU; (iii) classical K. pneumoniae, not lethal at a CI of 10^7^ CFU. Quantitative siderophore and mucoviscosity assays differentiated _fv_hvKp and _pv_hvKp strains from cKp strains but were unable to differentiate between the _fv_hvKP and _pv_hvKP strain cohorts. However, SQ challenge of CD1 mice and intraperitoneal (IP) challenge of CD1 and BALB/c mice, but not C57BL/6 mice, were able to discriminate between an _fv_hvKp and a _pv_hvKp strain; SQ challenge of CD1 mice may have the greatest sensitivity. cKp was differentiated from hvKp both by SQ challenge of CD1 mice and IP challenge of all three mouse strains. These data identify a means to define the relative virulence of hvKP strains. It remains unclear whether the observed differences of hvKp virulence in mice translates to human infection. However, these data can be used to sort random collections of K. pneumoniae strains into _fv_hvKp and _pv_hvKp strain cohorts and assess for differences in clinical manifestations and outcomes.

**IMPORTANCE** The pathogenic potential of hvKp strains is primarily mediated by a large virulence plasmid. The minimal set of genes required for the full expression of the hypervirulent phenotype is undefined. A number of reports describe hvKp strains possessing only a portion of the virulence plasmid; the clinical consequences of this are unclear. Therefore, the goal of this report was to determine whether virulence among hvKp strains varied and, if so, how to best identify the relative virulence of hvKp isolates. Data demonstrate hvKp pathogenic potential varies in CD1 and BALB/c murine infection models. In contrast, measurements of siderophore production and mucoviscosity were unable to discriminate the differences in hvKp isolate virulence observed in mice. This information can be used in future studies to determine the mechanisms responsible for differences between fully virulent hvKp and partially virulent hvKp and whether the differences observed in mice translate to disease in humans.

## INTRODUCTION

Klebsiella pneumoniae is widely known for its propensity to cause health care-associated and hospital-acquired infections (HAI) and has become increasingly resistant to antimicrobials (1, 2). However, it is less appreciated that two distinct pathotypes are currently circulating, termed classical K. pneumoniae (cKp) and hypervirulent K. pneumoniae (hvKp) (3), and selected epidemiologic, clinical, and genetic features distinguish these pathotypes. cKp is primarily an opportunistic pathogen that causes HAI in individuals with comorbidities; pneumonia, urinary tract infection, or surgical site infection are common infectious syndromes (4). In stark contrast, hvKp usually causes community-acquired infection (CAI), often in otherwise healthy hosts (3). A monomicrobial hepatic abscess in the absence of biliary disease and community-acquired pneumonia are the most commonly recognized syndromes; however, equally canonical features are the development of multiple sites of infection, several of which are unusual for *Klebsiella* in a healthy host such as meningitis, necrotizing fasciitis, epidural abscess, splenic, prostatic, or brain abscess, and endophthalmitis (3).

Initial studies established that the genotype that conferred hvKp’s hypervirulent phenotype was associated with the presence of a large >180-kDa plasmid on which hvKp-specific virulence factors resided (5–7). Genotypic biomarkers can be used to accurately identify hvKp strains and an inferred increase in virulence relative to cKp strains. The possession of the combination of the *iucA*, *iroB*, *peg-344*, _p_*rmpA*, and _p_*rmpA2* genes which reside on the canonical hvKp virulence plasmid, albeit imperfectly, most accurately predicts whether a strain possesses the hypervirulent phenotype. This result was validated in the outbred CD1 murine infection model, which was highly discriminatory for differentiating hvKp-rich and cKp-rich strain cohorts that were developed based on clinical criteria from human infection (8). Subsequently, a number of studies have described hvKp isolates that possess some, but not all, of these biomarkers and/or plasmids that contain only a portion of the genomic content present in pK2044 or pLVPK (9–13). Hybrid plasmids that consist of antimicrobial resistance genes and a fraction of the hvKp-specific virulence plasmid have also been described (14–16). However, it remains unclear which combination of genetic elements residing on the hvKp-specific virulence plasmid is needed for the full expression of the hypervirulent phenotype, the relative importance of individual elements, and whether these differences affect the pathogenic potential of a given hvKp strain.

Phenotypic markers have also been used as a means to identify the hypervirulent phenotype. These markers include a variety of *in vitro* assays, infection models, and the clinical syndrome. The string test, which is a qualitative test for mucoviscosity, is not optimally accurate for differentiating hvKp strains from cKp strains (3, 8). Neutrophil and complement-mediated bactericidal activity are also unable to reliably differentiate hvKp from cKp strains (17–20). Likewise, although capsule types have also been proposed as a means to identify hvKp strains, both cKp and hvKp strains can possess a given capsule type (3). However, the production of >30 μg/ml of siderophores in K. pneumoniae has been shown to strongly correlate with lethality in the outbred CD1 murine infection model (8), but this assay is burdensome and not routinely utilized. Alternatively, the phenotype of virulence in infection models is also used. The Galleria mellonella infection model has been commonly employed for this purpose, but it is unable to reliably differentiate hvKp from cKp strains (20–23). Although the clinical syndrome of invasive infection in healthy individuals from the community may be highly suggestive that the offending pathogen is an hvKp isolate, this distinction cannot always be made, especially with the recognition that hvKp can cause health care-associated and nosocomial infection (9, 24, 25). The most accurate phenotypic means to differentiate hvKp from cKp strains has been assessing virulence in mouse infection models such as subcutaneous (SQ) challenge of outbred CD1 mice (8). The accuracy of other mouse strains and challenge routes has been less rigorously assessed.

Data from our group, and others, have suggested that not all hvKp strains are equally virulent in murine infection models (8, 26). Therefore, the goal of this study was to establish whether a virulence spectrum of hypervirulent K. pneumoniae exists, and if so, the value of siderophore production, mucoviscosity, and various murine infection models for identifying differences in hvKP pathogenic potential. The data generated established that a virulence spectrum exists for hvKp strains when assessed in CD1 and BALB/c mice, but not C57BL/6 mice. Unfortunately, quantitative total siderophore production and quantitative mucoviscosity assays were not able to discern the differences in pathogenic potential of hvKp strains identified in mice. Whether the observed differences of hvKp virulence in mice translates to human infection is presently unknown but can now be elucidated in future studies.

## RESULTS

### Identification of the _fv_hvKp, _pv_hvKp, and cKp strain cohorts.

The CD1 subcutaneous challenge (SQ) infection model has been validated for differentiating hvKp from cKp pathotypes, and it is extraordinarily sensitive as evidenced by at least a 5-log-unit window for detecting differences in lethality after bacterial challenge with fully virulent hvKp and cKp strains. Therefore, this model was initially used to assess for potential differences in virulence between hvKp strains (8). Our hvKp strain library (*n* = 100) and a collection of isolates from the Walter Reed Army Institute of Research Multidrug-Resistant Organism Repository and Surveillance Network (MRSN) that were identified by possessing all of the hvKp-specific biomarkers *iucA*, *iroB*, _p_*rmpA*, _p_*rmpA2*, and *peg-344* (*n* = 13) underwent screening. The following three K. pneumoniae strain cohorts were defined by their phenotype after SQ challenge of outbred CD1 mice; fully virulent hvKp strains (_fv_hvKP) were lethal at a challenge inoculum (CI) of ≤10^3^ CFU; partially virulent hvKp strains (_pv_hvKp) were lethal at a CI >10^3^ to 10^7^ CFU; and classical K. pneumoniae (cKp) were not lethal at a CI of 10^7^ CFU. Of the 100 hvKp strains in our strain library that consists of isolates from otherwise healthy, ambulatory patients with a clinical syndrome of tissue invasive infection (e.g., hepatic and extrahepatic abscesses, necrotizing fasciitis, or endophthalmitis), 11/100 (11%) possessed a _pv_hvKP phenotype. Of the 13 MRSN isolates, 7/13 (54%) possessed a _pv_hvKP phenotype. For this study, 15 _fv_hvKp strains, 14 _pv_hvKp strains, and 15 cKp strains were chosen for further study. Quantitative lethality data for these strains is listed in Table S1 in the supplemental material. All _fv_hvKp strains and 13/14 _pv_hvKp strains (hvKp42 lacks *iucA* and *rmpA*) possess all of the hvKp-specific virulence plasmid genes *iucA*, *iroB*, _p_*rmpA*, _p_*rmpA2*, and *peg-344*, which have been shown to accurately identify hvKp strains (8), whereas none of the cKp strains possessed these biomarkers (Table S1). hvKp2 (_fv_hvKp), hvKp94 (_pv_hvKp), and cKp1 (cKp) were selected for additional detailed studies in CD1, BALB/c, and C57BL/6 mice. These data support that not all hvKp strains share a similar pathogenic potential in mice.

10.1128/mSphere.00045-21.1 TABLE S1 Strains used in this study. Download Table S1, XLSX file, 0.02 MB. Copyright © 2021 Russo et al. 2021 Russo et al. https://creativecommons.org/licenses/by/4.0/ This content is distributed under the terms of the Creative Commons Attribution 4.0 International license.

### SQ challenge of CD1 mice is able to differentiate hvKp2 (_fv_hvKp) from hvKp94 (_pv_hvKp).

**FIG 1:**
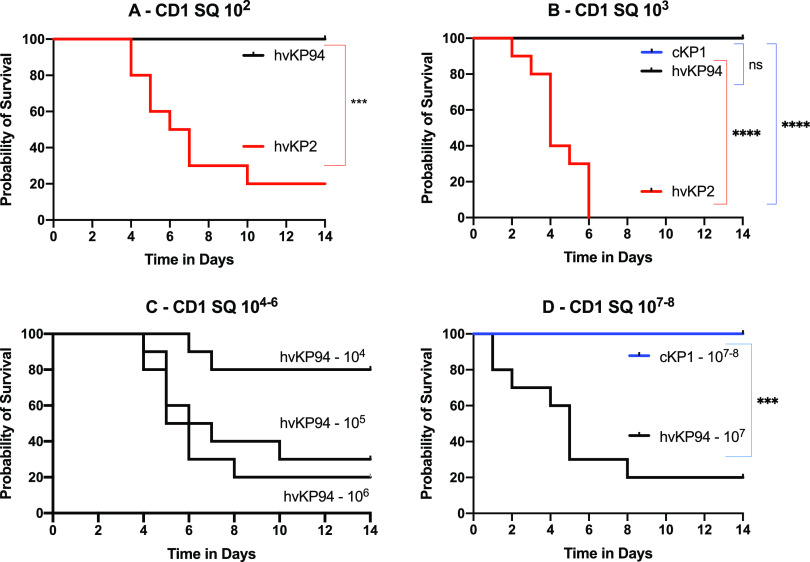
Survival of outbred CD1 mice after subcutaneous (SQ) challenge with hvKp2, hvKp94, and cKp1. (A) The mice were challenged with 2.6 × 10^2^ CFU of hvKp2 and 2.75 × 10^2^ CFU of hvKp94. (B) The mice were challenged with 2.8 × 10^3^ CFU of hvKp2, 3.15 × 10^3^ CFU of hvKp94, and 2.58 × 10^3^ CFU of cKp1. (C) The mice were challenged with 2.85 × 10^4^ CFU of hvKp94, 3.15 × 10^5^ CFU of hvKp94, and 2.85 × 10^6^ CFU of hvKp94. (D) The mice were challenged with 2.48 × 10^7^ CFU of hvKp94, 2.5 × 10^7^ CFU of cKp1, and 3.2 × 10^8^ CFU of cKp1. Strains were grown overnight in LB medium. An *in extremis* state or death was scored as nonsurvival. Total *n* = 10 (*n* = 5 in each of two independent experiments) for each titer for each strain. Values that are significantly different by the log rank (Mantel-Cox) test are indicated as follows: for panel A, ***, *P* = 0.0003; for panel B, ****, *P* < 0.0001; and for panel D, ***, *P* = 0.0003. ns, not significantly different.

CD1 mice underwent SQ challenge with the _fv_hvKP strain hvKp2 and the _pv_hvKp strain hvKp94. hvKp2 was significantly more lethal after SQ challenge with approximately 10^2^ to 10^3^ CFU compared to hvKp94 at similar titers; resultant mortality rates for these CIs were 80% and 100% for hvKP2 compared to 0% and 0% for hvKP94, respectively (Fig. 1A and B and Table 1). SQ challenge with approximately 10^4^ to 10^7^ CFU of hvKp94 resulted in mortality rates of 20%, 70%, 80%, and 80, respectively. Because a 100% mortality rate was predicted based on the 10^3^ CFU challenge data, mice were not challenged with 10^4-7^ CFU of hvKp2 for comparative purposes, since this was deemed inappropriate. Exact titers and *P* values are listed in Tables S3 and 4, respectively. These data further support that a virulence spectrum exists for hvKp and that SQ challenge of CD1 mice can clearly distinguish between _fv_hvKp and _pv_hvKp strains.

**TABLE 1:** Mortality rates of CD1, BALB/c, and C57BL/6 mice after challenge with cKp1, hvKp2, and hvKp94

Mouse	Infection^*a*^	Strain	Mortality rate (%) for mice challenged with the following CFU:							LD_50_^*b*^
			10^2^	10^3^	10^4^	10^5^	10^6^	10^7^	10^8^	
CD1	SQ	cKp1		0				0	0	UC
		hvKp2	80	100						UC
		hvKp94	0	0	20	70	80	80		1.2 × 10^5^
CD1	IP	cKP1		0	0	0	0	0	60	2.38 × 10^8^
		hvKp2	40	60	70	100				1.12 × 10^3^
		hvKp94	0	0	0	50				UC
BALB/c	IP	cKP1		0	0	0	0	0	60	2.61 × 10^8^
		hvKp2	30	70	90	100				7.98 × 10^2^
		hvKp94	0	0	0	30	90	100		5.44 × 10^5^
C57BL/6	IP	cKp1		0	0	0	0	0	80	2.34 × 10^8^
		hvKp2	20	40	70	90				4.56 × 10^3^
		hvKp94	0	0	50	80	100			3.19 × 10^4^

a SQ, subcutaneous challenge; IP, intraperitoneal challenge.

b LD_50_, 50% lethal dose; UC, unable to calculate.

10.1128/mSphere.00045-21.3 TABLE S3 Challenge inocula for animal experiments. Download Table S3, XLSX file, 0.01 MB. Copyright © 2021 Russo et al. 2021 Russo et al. https://creativecommons.org/licenses/by/4.0/ This content is distributed under the terms of the Creative Commons Attribution 4.0 International license.

### IP challenge of BALB/c and CD1 mice, but not C57BL/6 mice, is able to differentiate hvKp2 (_fv_hvKp) from hvKp94 (_pv_hvKp).

**FIG 2:**
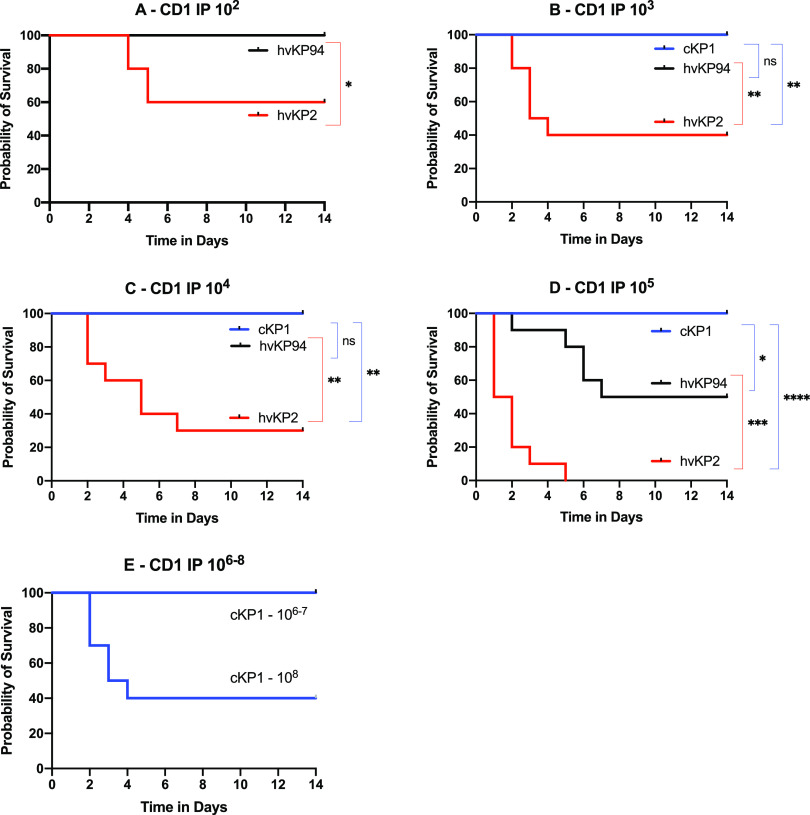
Survival of outbred CD1 mice after intraperitoneal (IP) challenge with hvKp2, hvKp94, and cKp1. (A) The mice were challenged with 2.65 × 10^2^ CFU of hvKp2 and 2.90 × 10^2^ CFU of hvKp94. (B) The mice were challenged with 3.96 × 10^3^ CFU of hvKp2, 2.73 × 10^3^ CFU of hvKp94, and 2.05 × 10^3^ CFU of cKp1. (C) The mice were challenged with 3.96 × 10^4^ CFU of hvKp2, 2.73 × 10^4^ CFU of hvKp94, and 2.05 × 10^4^ CFU of cKp1. (D) The mice were challenged with 3.96 × 10^5^ CFU of hvKp2, 2.90 × 10^5^ CFU of hvKp94, and 1.45 × 10^5^ CFU of cKp1. (E) The mice were challenged with 1.45 × 10^6^ CFU of cKp1, 1.45 × 10^7^ CFU of cKp1, and 2.5 × 10^8^ CFU of cKp1. Strains were grown overnight in LB medium. An *in extremis* state or death was scored as nonsurvival. Total *n* = 10 (*n* = 5 in each of two independent experiments) for each titer for each strain. Values that are significantly different by the log rank (Mantel-Cox) test are indicated as follows: for panel A, *, *P* = 0.03; for panel B, **, *P* = 0.004; for panel C, **, *P* = 0.001; for panel D, *, *P* = 0.01, ***, *P* = 0.0001, and ****, *P* < 0.0001. ns, not significantly different.

**FIG 3:**
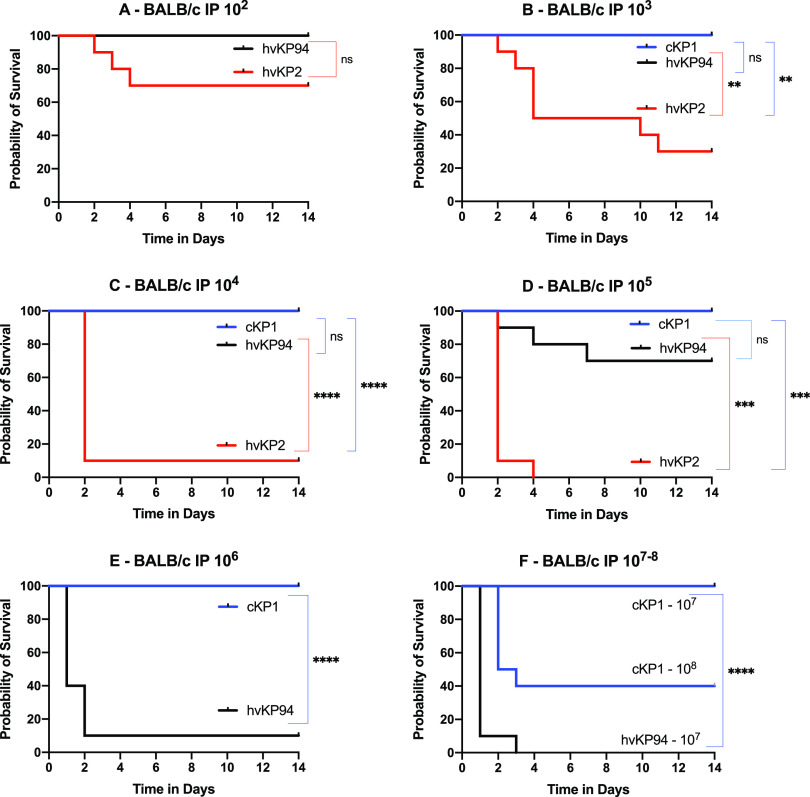
Survival of BALB/c mice after intraperitoneal (IP) challenge with hvKp2, hvKp94, and cKp1. (A) The mice were challenged with 2.60 × 10^2^ CFU of hvKp2 and 2.75 × 10^2^ CFU of hvKp94. (B) The mice were challenged with 2.35 × 10^3^ CFU of hvKp2, 2.80 × 10^3^ CFU of hvKp94, and 1.32 × 10^3^ CFU of cKp1. (C) The mice were challenged with 2.35 × 10^4^ CFU of hvKp2, 2.80 × 10^4^ CFU of hvKp94, and 1.32 × 10^4^ CFU of cKp1. (D) The mice were challenged with 2.35 × 10^5^ CFU of hvKp2, 2.95 × 10^5^ CFU of hvKp94, and 1.32 × 10^5^ CFU of cKp1. (E) The mice were challenged with 2.78 × 10^6^ CFU of hvKp94 and 1.93 × 10^6^ CFU of cKp1. (F) The mice were challenged with .93 × 10^7^ CFU of cKp1 and 2.73 × 10^8^ CFU of cKp1. Strains were grown overnight in LB medium. An *in extremis* state or death was scored as nonsurvival. Total *n* = 10 (*n* = 5 in each of two independent experiments) for each titer for each strain. Values that are significantly different by the log rank (Mantel-Cox) test are indicated as follows: for panel B, **, *P* = 0.001; for panel C, ****, *P* < 0.0001; for panel D, ***, *P* = 0.0001 (cKp1 compared to hvKp2) and ***, *P* = 0.0002 (hvKp2 compared to hvKp94); for panel E, ****, *P* < 0.0001; for panel F, ****, *P* < 0.0001. ns, not significantly different.

**FIG 4:**
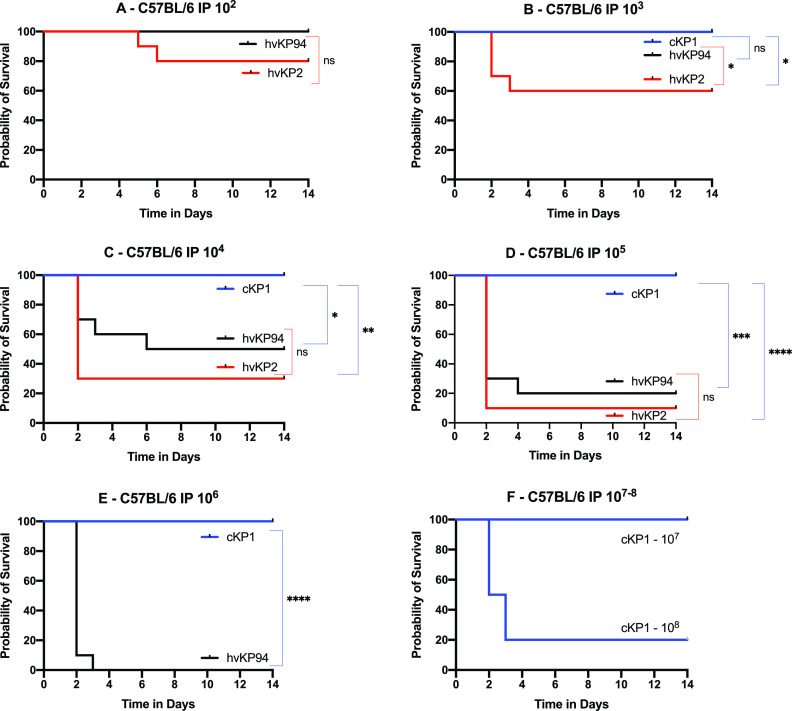
Survival of C57BL/6 mice after intraperitoneal (IP) challenge with hvKp2, hvKp94, and cKp1. (A) The mice were challenged with 2.60 × 10^2^ CFU of hvKp2 and 2.75 × 10^2^ CFU of hvKp94. (B) The mice were challenged with 2.35 × 10^3^ CFU of hvKp2, 2.8 × 10^3^ CFU of hvKp94, and 1.32 × 10^3^ CFU of cKp1. (C) The mice were challenged with 2.35 × 10^4^ CFU of hvKp2, 2.95 × 10^4^ CFU of hvKp94, and 1.32 × 10^4^ CFU of cKp1. (D) The mice were challenged with 2.35 × 10^5^ CFU of hvKp2, 2.95 × 10^5^ CFU of hvKp94, and 1.32 × 10^5^ CFU of cKp1. (E) The mice were challenged with 2.78 × 10^6^ CFU of hvKp94 and 1.93 × 10^6^ CFU of cKp1. (F) The mice were challenged with 1.93 × 10^7^ CFU of cKp1 and 2.73 × 10^8^ CFU of cKp1. Strains were grown overnight in LB medium. An *in extremis* state or death was scored as nonsurvival. Total *n* = 10 (*n* = 5 in each of two independent experiments) for each titer for each strain. Values that are significantly different by the log rank (Mantel-Cox) test are indicated as follows: for panel B, *, *P* = 0.03; for panel C, *, *P* = 0.01, **, *P* = 0.001; for panel D, ***, *P* = 0.0003, ****, *P* < 0.0001; for panel E, ****, *P* = 0.0001. ns, not significantly different.

Intraperitoneal (IP) challenge of C57BL/6 or BALB/c mice is commonly used to determine the relative virulence of K. pneumoniae. Therefore, these mouse strains underwent challenge with the _fv_hvKP strain hvKp2 and the _pv_hvKp strain hvKp94 to determine whether IP challenge of these mice would be able to differentiate _fv_hvKp and _pv_hvKp strains. C57BL/6, BALB/c, and CD1 mice underwent IP challenge with hvKP2 (_fv_hvKp) and hvKP94 (_pv_hvKp) (Fig. 2, 3, and 4 and Table 1). When CD1 and BALB/c mice were challenged IP with hvKP2 and hvKP94, significant differences in mortality rates were observed over multiple CIs (Fig. 2 and 3 and Table 1). For CD1 mice, hvKP2 was significantly more lethal after IP challenge with approximately 10^2^ to 10^5^ CFU compared to hvKP94 at similar titers; resultant mortality rates for these CIs were 40%, 60%, 70%, and 100% for hvKP2 compared to 0%, 0%, 0%, and 50% for hvKP94, respectively (Fig. 2A to D and Table 1). For BALB/c mice, hvKP2 trended (*P* = 0.067) more lethal after IP challenge with approximately 10^2^ CFU and was significantly more lethal with approximately 10^3^ to 10^5^ CFU compared to hvKP94 at similar titers; resultant mortality rates for these CIs were 70%, 90%, and 100% for hvKP2 compared to 0%, 0%, and 30%, for hvKP94, respectively (Fig. 3A to D and Table 1). By contrast, for C57BL/6 mice, a significant difference in lethality was observed only after IP challenge with approximately 10^3^ CFU of hvKp2 compared to hvKp94 at a similar titer; the resultant mortality rates for this CI were 40% and 0%, respectively (Fig. 4B and Table 1). Exact titers and *P* values are listed in Tables S3 and 4, respectively. These data support that IP challenge of CD1 and BALB/c mice, but not C57BL/c mice, can clearly distinguish between _fv_hvKp and _pv_hvKp strains.

### SQ challenge of CD1 mice may be more sensitive for differentiating _fv_hvKp from _pv_hvKp strains compared to IP challenge of CD1 and BALB/c mice.

**FIG 5:**
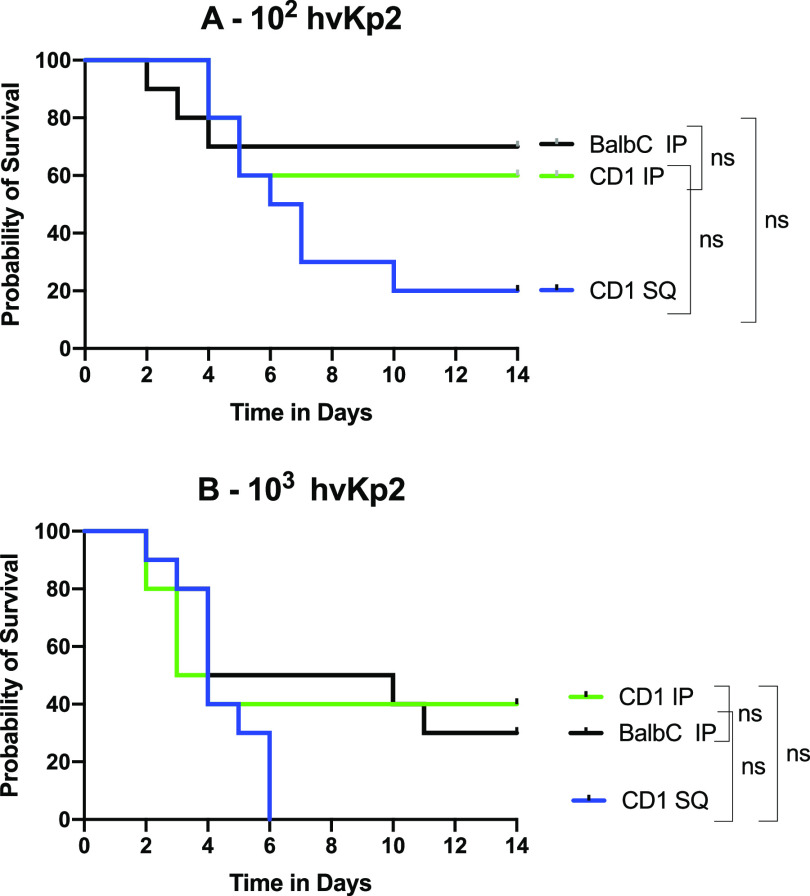
Comparison of CD1 mice challenged SQ with hvKp2 to CD1 and BALB/c mice challenged IP with hvKp2. (A) The CD1 mice were challenged with 2.6 × 10^2^ CFU of hvKp2 SQ and 2.65 × 10^2^ CFU of hvKp2 IP, and the BALB/c mice were IP challenged with 2.6 × 10^2^ CFU of hvKp2. (B) The CD1 mice were challenged with 2.80 × 10^3^ CFU of hvKp2 SQ and 3.96 × 10^3^ CFU of hvKp2 IP, and the BALB/c mice were IP challenged with 2.35 × 10^3^ CFU of hvKp2. Strains were grown overnight in LB medium. An *in extremis* state or death was scored as nonsurvival. Total *n* = 10 (*n* = 5 in each of two independent experiments) for each titer for each strain. ns, not significantly different.

Although both SQ and IP challenge of CD1 mice and IP challenge of BALB/c mice were able to identify significant differences in virulence, as measured by mortality rates between hvKp2 (_fv_hvKp) and hvKp94 (_pv_hvKp), SQ challenge was more numerically discerning than IP challenge (Fig. 5 and Table 1). Mortality rates for CD1 mice after SQ challenge with approximately 10^2^ to 10^3^ CFU of hvKP2 were 80% and 100%, respectively (Fig. 5 and Table 1) compared to mortality rates after IP challenge of CD1 mice of 40% (*P* = 0.158) and 60% (*P* = 0.381), respectively (Fig. 5 and Table 1), and to mortality rates after IP challenge of BALB/c mice of 30% (*P* = 0.095) and 70% (*P* = 0.077), respectively (Fig. 5 and Table 1). No mortality was observed for hvKp94 at similar CIs in CD1 mice challenged SQ and CD1 and BALB/c mice challenged IP. The 100% percent morality rate seen after SQ challenge of CD1 mice with approximately 10^3^ CFU of hvKp2 was not observed after IP challenge in CD1 and BALB/c mice until a CI of approximately 10^5^ CFU was administered (Table 1). Taken together, these data suggest that SQ challenge of CD1 mice may be more sensitive for differentiating _fv_hvKp from _pv_hvKp strains than IP challenge of CD1 or BALB/c mice.

### IP challenge of BALB/c, CD1, and C57BL/6 mice or SQ challenge of CD1 mice is able to differentiate hvKp from cKp strains.

In all tested mouse strains and challenge routes, no mortality occurred when challenged with ≤1 × 10^7^ CFU of cKP1 (Fig. 1, 2, 3, and 4 and Table 1). Due to the enhanced virulence of the _fv_hvK strain hvKp2, lethality was readily noted when low CIs of 10^2^ to 10^3^ CFU were used, whereas for the less virulent _pv_hvK strain hvKp94, this was not observed consistently until CIs of ≥10^5^ to 10^6^ CFU were used. Interestingly, mortality rates of 60%, 60%, and 80% were observed when CD1, BALB/c, or C57BL/6 mice were challenged IP with approximately 10^8^ CFU of cKP1. By contrast, a 0% mortality rate was observed when CD1 mice were challenged SQ with 3.2 × 10^8^ CFU of cKp1. Whether this was due to a direct bacterial effect and/or the host inflammatory response induced by this high IP CI is unclear. These data support that SQ challenge of CD1 mice or IP challenge of CD1, BALB/c, or C57BL/c mice can distinguish between hvKp and cKp strains.

### Quantitative siderophore or mucoviscosity assays are able to accurately differentiate hvKp from cKp strains but are unable to differentiate _fv_hvKp strains from _pv_hvKp strains.

Although the murine CD1 SQ challenge and the CD1 and BALB/c IP challenge infection models can accurately differentiate _fv_hvKp from _pv_hvKp strains, their use is not pragmatic for the clinical microbiology laboratory or for the study of large numbers of strains. Further, the development of alternatives to the use of animals is desirable when possible. Therefore, we assessed whether the phenotypes of quantitative total siderophore production and mucoviscosity could be used to accurately differentiate _fv_hvKp from _pv_hvKp strains.

**FIG 6:**
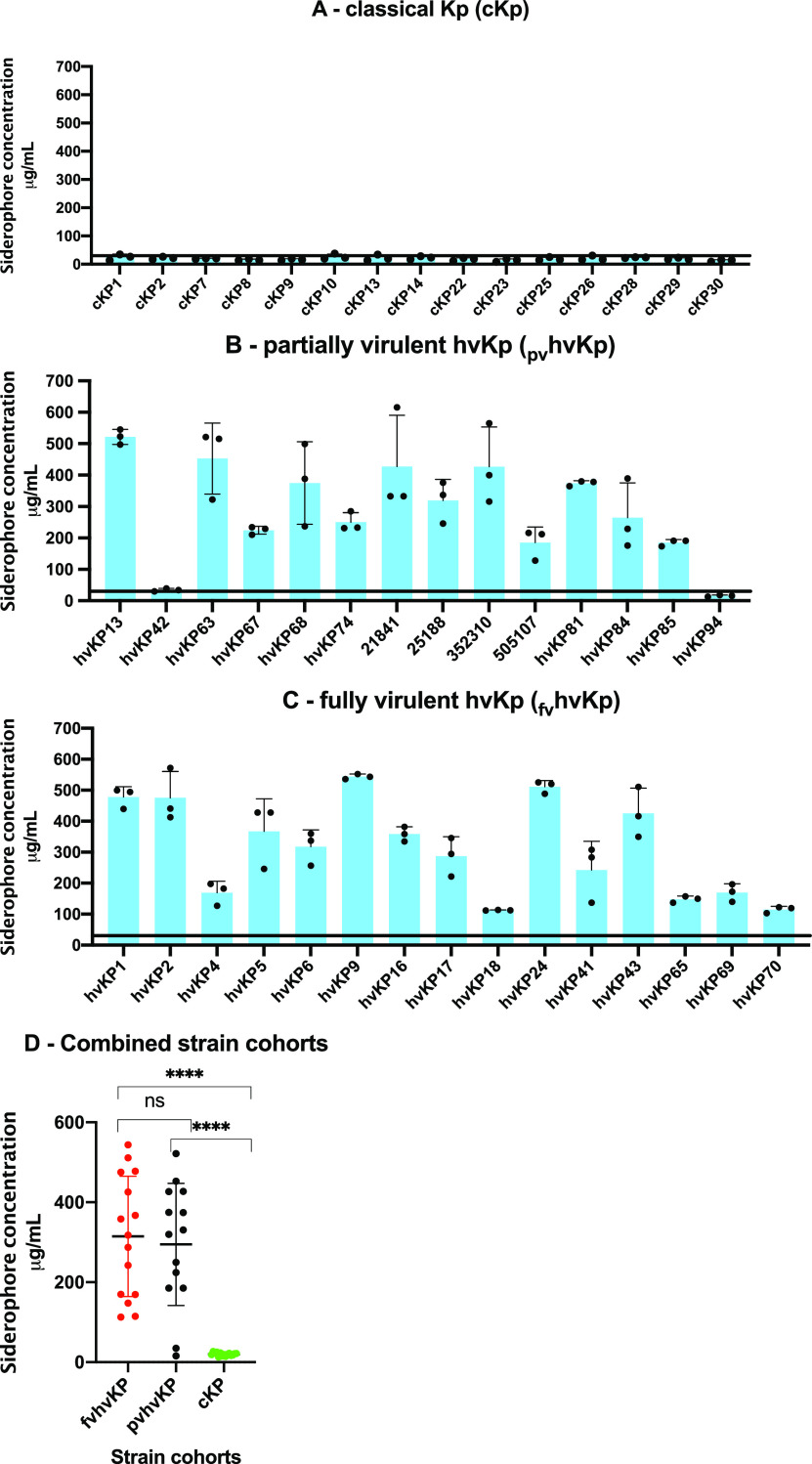
Quantitative siderophore production of cohorts of classical K. pneumoniae (cKp) strains, partially virulent hypervirulent K. pneumoniae (_pv_hvKp) strains, and fully virulent hypervirulent K. pneumoniae (_fv_hvKp) strains. (A) cKp strain cohort; (B) _pv_hvKp strain cohort; (C) _fv_hvKp strain cohort; (D) All cohorts. Bacterial strains were grown in iron-chelated M9 minimal medium plus Casamino Acids (c-M9-CA). The horizontal line marks 30 μg/ml. Quantitative siderophore production culture supernatants were assessed from culture supernatants using the chromeazurol S dye assay as described previously (36). A total of three independent assays were performed on different days, and the results were reported as means ± SD. ****, *P* < 0.0001 by Mann-Whitney test. ns, not significantly different.

**(i) Quantitative siderophore assay.** Previous data have shown that total siderophore production of >30 μg/ml is able to accurately differentiate between hvKp and cKp strains (8). Therefore, the next logical step was to determine whether total siderophore production could differentiate _fv_hvKp from _pv_hvKp strains. To accomplish this, three strain cohorts were assessed: (i) _fv_hvKp strains (*n* = 15), (ii) _pv_hvKp strains (*n* = 14), and (iii) cKp strains (*n* = 15) (Table S1). The mean siderophore concentration was 315 μg/ml ± 151 μg/ml, 295 μg/ml ± 153 μg/ml, and 20 μg/ml ± 4.1 μg/ml for the _fv_hvKp, _pv_hvKp, and cKp strain cohorts, respectively (_fv_hvKp compared to _pv_hvKp, *P* = 0.11; _fv_hvKp or _pv_hvKp compared to cKp, *P* < 0.0001). Results from individual strains are depicted in Fig. 6. All of the _fv_hvKp strains and 12/14 _pv_hvKp strains produced siderophore levels of >100 μg/ml. Although 2/14 _pv_hvKp strains produced siderophore levels of 15.85 ± 2.57 and 34.51 ± 4.9, these data do not support the use of quantitative siderophore measurement as a means to differentiate _fv_hvKp strains from _pv_hvKp strains.

**FIG 7:**
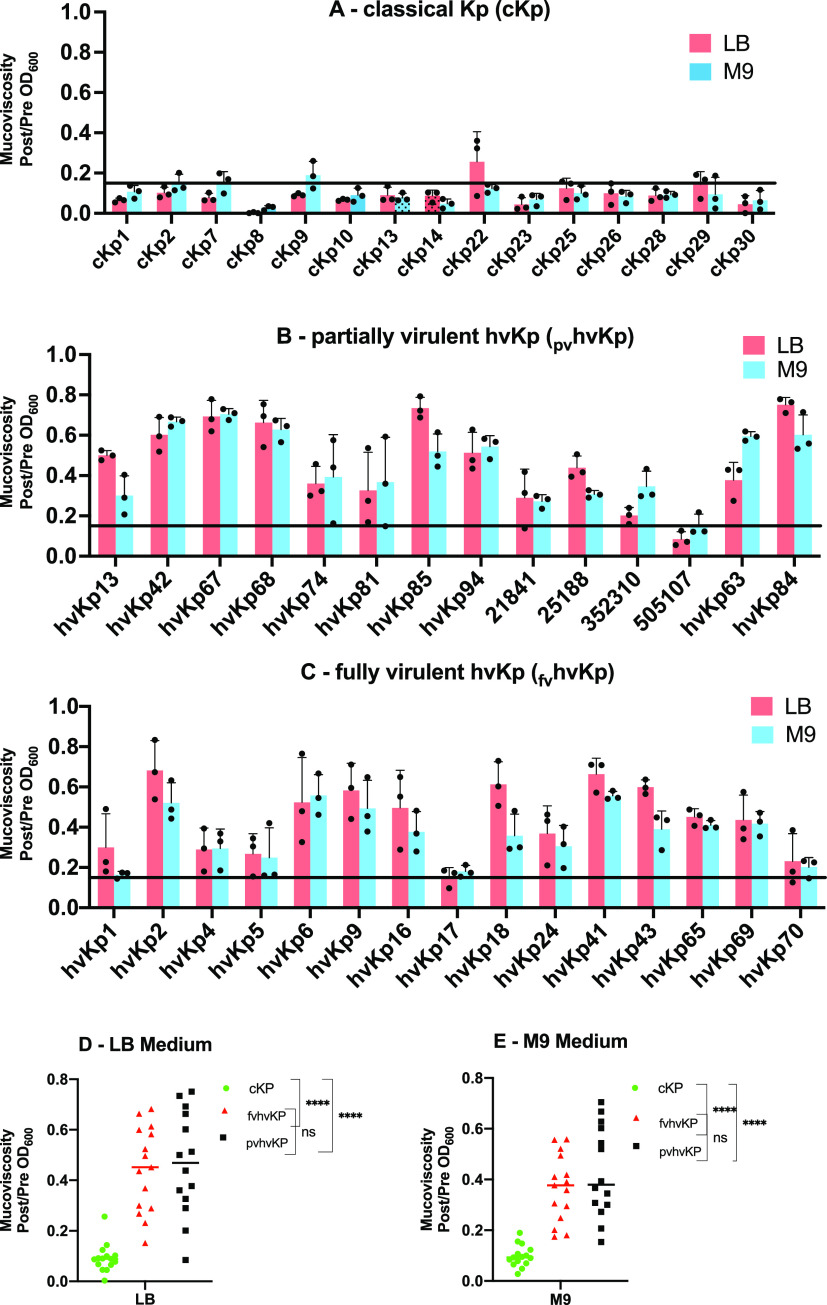
Mucoviscosity of cohorts of classical K. pneumoniae (cKp) strains, partially virulent hypervirulent K. pneumoniae (_pv_hvKp) strains, and fully virulent hypervirulent K. pneumoniae (_fv_hvKp) strains. Bacterial strains were grown in either lysogeny broth (LB) or iron-chelated M9 minimal medium plus added trace elements (c-M9-te). The culture OD_600_ was normalized to 1.0 and then centrifuged at 1,000 × *g* for 5 min. Mucoviscosity was recorded as the post/prespin OD_600_ ratio. (A) cKp strain cohort; (B) _pv_hvKp strain cohort; (C) _fv_hvKp strain cohort; (D) all cohorts grown in LB; (E) all cohorts grown in c-M9-te. The horizontal line marks 0.15. Three independent assays were performed on different days for each strain, and the data were reported as means ± SD. ****, *P* < 0.0001 by two-way ANOVA.

**(ii) Quantitative mucoviscosity assay.** Mucoviscosity, which is due to RmpA regulated production of RmpD, is a phenotype that is associated with hvKp strains (27–29). Therefore, we performed a quantitative mucoviscosity assay in both lysogeny broth (LB) and c-M9-te (iron-chelated M9 minimum medium plus added trace elements) broth to assess the _fv_hvKp, _pv_hvKp, and cKp strain cohorts. The mean mucoviscosity (post/prespin optical density at 600 nm [OD_600_] ratio) values were 0.44 ± 0.17, 0.47 ± 0.21, and 0.09 ± 0.06 for the _fv_hvKp, _pv_hvKp, and cKp strain cohorts, respectively, when grown in LB and 0.37 μg/ml ± 0.13, 0.46 ± 0.18, and 0.10 ± 0.04 when grown in c-M9-te (_fv_hvKp compared to _pv_hvKp, *P* = 0.50; _fv_hvKp or _pv_hvKp compared to cKp, *P* < 0.0001). Results from individual strains are depicted in Fig. 7. All 15 of the _fv_hvKp and _pv_hvKp strains had a post/prespin OD_600_ ratio of >0.15 when grown in c-M9-te broth; 15/15 and 13/14 of the _fv_hvKp and _pv_hvKp strains had a post/prespin OD_600_ ratio of >0.15 when grown in LB. By contrast, 1/15 and 2/15 cKp strains had a post/prespin OD_600_ ratio of >0.15 when grown in LB and c-M9-te broth, respectively. Interestingly, the mean mucoviscosity values for the index _pv_hvKp strain hvKp94 were 0.51 ± 0.10 and 0.54 ± 0.05 when grown in LB and c-M9-te broth, respectively, similar to _fv_hvKp strains. These data demonstrate that the quantitative mucoviscosity assay on average is able to differentiate hvKp from cKp strains but is unable to differentiate _fv_hvKp from _pv_hvKp strains.

## DISCUSSION

Differentiating cKp from hvKp purely on clinical grounds can be challenging. There is an overlap for selected clinical syndromes, and this becomes more challenging with the recognition that hvKp is causing health care-associated and nosocomial infections similar to cKp (9, 24, 25). Making this distinction has important management implications for fully virulent hvKp strains, for example, vigilance for endophthalmitis, consideration of searching for occult abscess, the use of the largest bore drainage catheter possible, an awareness of propensity for recurrence/relapse, and perhaps hvKp-specific infection control practices (3, 30, 31). Presently, an FDA-approved test for use in the clinical microbiology laboratory is not available to distinguish hvKp strains from cKp strains. However, the presence of *iucA*, *iroB*, *peg-344*, _p_*rmpA*, and _p_*rmpA2* or siderophore production of >30 μg/ml have been shown to accurately differentiate the hvKp and cKp pathotypes (8). Further, albeit limited, data from this report support that a quantitative mucoviscosity assay also holds promise for distinguishing hvKp from cKp. Although impractical for the clinical microbiology laboratory, SQ challenge of CD1 mice is sensitive and accurate for distinguishing the hvKp and cKp pathotypes (8).

Data generated by our group while developing assays to differentiate hvKp from cKp strains, and by others, have suggested that not all hvKp strains are equally virulent in murine infection models (8, 26). Therefore, the goal of this report was to determine whether virulence among hvKp strains varied and, if so, how to best identify the relative virulence of hvKp isolates. Data presented demonstrate that not all hvKp strains share a similar pathogenic potential when assessed by SQ and IP challenge in CD1 mice and by IP challenge of BALB/c mice. Unfortunately, assays that quantitatively measured total siderophore production and mucoviscosity were unable to distinguish between fully and partially virulent hvKp isolates.

It is important to note that it remains unclear whether the observed differences of hvKp virulence in mice translate to human infection. To best answer this question, a random collection of K. pneumoniae strains for which clinical data are available would need to be sorted into _fv_hvKp, _pv_hvKp, and cKp strain cohorts and assessed for potential differences in clinical manifestations and outcomes. Key clinical metrics to measure would be the relative ability of each strain cohort to infect multiple sites, metastatically spread, and/or mediate infectious syndromes not usually observed for cKp strains such as central nervous system infection, endophthalmitis, or necrotizing fasciitis. Understanding the clinical behavior of _pv_hvKp strains in humans could have important implications for management and infection control (3, 30, 31).

The clinical data available for the _pv_hvKp strains in our collection does not resolve this issue. First, these isolates were not random. Ten isolates were part of the hvKp-rich strain cohort that was used to identify biomarkers to define hvKp (8), and inclusion criteria were based on (i) community acquisition in an otherwise healthy host, (ii) multiple sites of infection or metastatic spread, and (iii) unusual sites of infection for cKp such as endophthalmitis, meningitis, necrotizing fasciitis, nonhepatic abscess (8). Four isolates were from a collection of multidrug-resistant and extremely drug-resistant (MDR-XDR) isolates obtained from military personnel, which were subsequently shown to be hvKp based on the presence of the biomarkers *iucA*, *iroB*, _p_*rmpA*, _p_*rmpA2*, and *peg-344*. Nonetheless, some information can be gleaned from this collection. Of 11 _pv_hvKp strains for which clinical data were available, 10 caused community-acquired infection. Only one of these strains (hvKp42) resulted in multiple sites of infection causing meningitis and brain abscess. However, this strain was from Taiwan and shipped unfrozen to the United States for study. Therefore, a genetic alteration due to handling and storage cannot be excluded. The _pv_hvKp strain hvKp94 caused prostatic abscesses, but it was likely directly introduced as a result of transrectal biopsy. Therefore, since _pv_hvKp strains can cause community-acquired infection in otherwise healthy hosts, clinical data support enhanced virulence compared to cKp strains, but it remains unclear whether they are equally virulent to _fv_hvKp strains and if differences exist, what the management and infection control implications would be, if any.

It is important to note that a knowledge gap exists on the relative virulence of strains having fewer than four or five of *iucA*, *iroB*, *peg-344*, _p_*rmpA*, and _p_*rmpA* biomarkers. Twenty-eight of 29 hvKp strains in this study possessed all five of these markers (*iucA and rmpA2* were absent in one strain), which is not surprising since they are usually linked on the canonical hvKp virulence plasmid (8). Preliminary data from our group that assessed strains with only one to three of these biomarkers in the CD1 SQ challenge model suggest that these strains are significantly less virulent than the _fv_hvKp and _pv_hvKp pathotypes in murine infection models. Additional studies are needed to establish where they stand on the virulence spectrum between _fv_hvKp and cKp and the potential clinical implications for human disease. Additionally, *iutA*, *iroE*, and _p_*rmpA* can be located within integrative and conjugative elements and may be less reliable markers for the hvKp hypervirulent phenotype since their presence does not necessarily predict the presence of the virulence plasmid (32).

The spectrum of virulence of hvKp strains can be defined via the use of murine infection models. In this study, we established that SQ challenge of CD1 mice or IP challenge of CD1 and BALB/c mice enabled the identification of hvKp strains with decreased virulence that we have termed _pv_hvKp; SQ challenge of CD1 mice may be the most sensitive model for discerning this difference. Importantly, IP challenge of C57BL/6 mice was poorly discriminatory, so not all mouse models are equal in this regard. Although the mechanisms responsible for these differences were not assessed, one could speculate that outbred mice are more resistant to infection, which in turn increases discriminatory power. Likewise, BALB/c mice possess a TH-2 bias, thereby resulting in increased resistance to extracellular pathogens such as K. pneumoniae, whereas C57BL/6 mice possess a TH-1 bias that results in decreased resistance to extracellular pathogens (18). The route of infection also appears to be important, which may be due to differences in site-specific host defenses. Further, IP challenge results in a more rapid bacteremia due to peritoneal fluid drainage via the lymphatics directly into the bloodstream, which may expedite clearance of lower CIs.

As demonstrated in this study of hvKP94 and other _pv_hvKp strains, the presence of the combination of *iucA*, *iroB*, *peg-344*, _p_*rmp*A, and _p_*rmpA2* does not necessarily predict the full hypervirulent pathotype, at least as defined in murine infection models (8, 26, 33). One explanation is that other, yet to be identified, virulence genes are needed for maximal virulence, and these genes may not be closely linked with *iucA*, *iroB*, *peg-344*, _p_*rmpA*, and _p_*rmpA2*. A genomic comparison of _fv_hvKp strains with _pv_hvKp strains may prove to be insightful for delineating the full pantheon of virulence factors requisite for expression of a complete hvKp phenotype.

It is certain that multiple different mechanisms could be responsible for decreased virulence in _pv_hvKp strains. The first would be the absence or decreased expression of defined virulence factors. The _pv_hvKp strain hvKp94 produced low levels of total siderophores. It has been previously shown that aerobactin accounts for the majority of siderophores produced by hvKp (34). Preliminary data did not demonstrate a genomic alteration in the aerobactin biosynthetic operon for hvKp94 that precluded production. Therefore, the mechanism(s) responsible for low siderophore production is of interest and may lend insight into the regulation of aerobactin production in hvKp.

This study has several limitations. The mouse breeds assessed were not exhaustive nor were the routes of infection. For example, pulmonary challenge was not studied. However, it was previously shown that lethality was similar after SQ and pulmonary challenge of CD1 mice with the _fv_hvKp strain hvKp1 (35). For this study, male mice were exclusively used; therefore, any potential gender-based differences would not have been identified. It would also be important to perform the quantitative mucoviscosity assay and *in vivo* studies on a greater number of _fv_hvKp, _pv_hvKp, and cKp strains. Studies on putative hvKp strains that do not possess the complete repertoire set of *iucA*, *iroB*, *peg-344*, _p_*rmpA*, and _p_*rmpA2* would also be of interest.

In summary, this report established that in murine infection models, not all hvKp strains are equally virulent. It remains unclear whether the partially virulent hvKp phenotype observed in mice translates to human disease. This will be important to determine since there are important differences in the management of infections caused by fully virulent hvKp compared to cKp.

## MATERIALS AND METHODS

### Bacterial strains.

The bacterial strains used in this study, their origin, sites of infection, and selected genotypic and phenotypic characteristics are listed in Table S1 in the supplemental material. A number of these strains have been previously reported (designated by reference 8 in Table S1) for which quantitative siderophore production and semiquantitative *in vivo* data were previously reported. For in-depth murine infection studies hvKp2, hvKp94, and cKp1 were used. hvKp2 (ST23, K1; *iucA*^+^ *iroB*^+^ *peg-344*^+^_p_*rmpA*^+^_p_*rmpA2*^+^) was isolated from the blood of a patient who presented from the community in Buffalo, New York, with bacteremia and endophthalmitis (8). hvKp94 (ST23, K1; *iucA*^+^ *iroB*^+^ *peg-344*^+^_p_*rmpA*^+^_p_*rmpA2*^+^) was isolated from the blood of a patient from Buffalo, New York, post-transrectal prostatic biopsy that was complicated by multiple prostatic abscesses. cKp1 (ST2424-1LV, K undefined [not K1, K2, K5, K20, K54, K57]; *iucA iroB peg-344*_p_*rmpA*_p_*rmpA2* not present) was isolated from the blood of a patient from Montreal, Quebec, Canada (8). All strains were stored at −80°C prior to use.

### Multiplex PCR assay for the identification of selected virulence genes.

Genomic DNA (gDNA) was extracted from 300 μl of a strain grown overnight in lysogeny broth (LB) (5 g yeast extract, 10 g tryptone, 10 g NaCl). Cells underwent centrifugation, and the resultant pellet was resuspended and incubated for 15 min in a Tris-EDTA (TE) lysing buffer containing 0.5% sodium dodecyl sulfate (SDS) and 0.16 mg/ml proteinase K, followed by a 30-min incubation at 37°C with DNase-free RNase (0.033 mg/ml). Next, 36 μl of 5 M sodium chloride was added, and the solution was vortexed vigorously and centrifuged (4°C, 11,500 × *g*, 10 min) to precipitate protein and cellular debris. The gDNA was extracted using isopropanol, followed by two washes with 70% ethanol before resuspension in 10 mM Tris (pH 8.5). DNA concentration and purity were assessed using a Nanodrop One spectrophotometer with a minimum requirement of a 260/280 ratio of ≥1.8. Multiplex PCR was performed using 2× Frogga *Taq* Mix, 12.5 ng gDNA, 0.1 nM each primer and nuclease-free water in a 10-μl reaction mixture. PCR conditions were as follows: 2 min at 95°C; 35 cycles, with 1 cycle consisting of 15 s at 95°C, 30 s at 60/53°C, and 1.5 min at 72°C, with a hold at 4°C. The PCRs were analyzed on a 1.5% agarose gel. Four multiplex PCRs were performed with the following primer pair combinations (Table S2): (i) *iucA* pair #1, *iroB*, *irp2*, and *gapA* (control for DNA integrity); (ii) *peg589* and *peg1631*; (iii) *peg-344* and *_p_rmpA*; (iv) *iucA* pair #2, *_p_rmpA2* and *terB*. The hypervirulent strain hvKP1 (8) served as a positive control, and water served as contamination control.

10.1128/mSphere.00045-21.2 TABLE S2 Multiplex PCR primers. Download Table S2, XLSX file, 0.01 MB. Copyright © 2021 Russo et al. 2021 Russo et al. https://creativecommons.org/licenses/by/4.0/ This content is distributed under the terms of the Creative Commons Attribution 4.0 International license.

### Quantitative siderophore assay.

For the quantitative siderophore assay, strains were grown individually overnight at 37°C in iron-chelated M9 minimal medium containing Casamino Acids (c-M9-CA) (35), and culture supernatants were assessed using the chromeazurol S dye assay as described previously (36). A minimum of three assays were performed, and the results were reported as means ± standard deviations (SD).

### Mucoviscosity assay.

Overnight cultures grown in LB broth were used to inoculate 10 ml of either LB or iron-chelated M9 minimum medium plus added trace elements (5 μg/ml CaCl_2_, 1 μg/ml CoCl_2_, 20 μg/ml MgCl_2_, 10 μg/ml MnCl_2_) (c-M9-te) to a starting OD_600_ of approximately 0.2. Pilot experiments compared the mucoviscosity of selected strains grown to either an OD_600_ of 1.0, grown for 6 h, or grown for 24 h. It was determined that the 24-h cultures demonstrated higher mucoviscosity values and allowed greater differentiation between strains; therefore, mucoviscosity was measured after growth for 24 h. Strains were grown shaking (275 rpm) for 24 h at 37°C, and the OD_600_ was normalized to 1.0 (prespin OD_600_) with either LB or c-M9-te minimal medium as appropriate. The OD_600_-adjusted culture (1.5 ml) was then centrifuged at 1,000 × *g* for 5 min. Seven hundred microliters was carefully withdrawn from the top of the supernatant without disturbing the pellet, and the OD_600_ was measured (postspin OD_600_). Mucoviscosity was recorded as the post/prespin OD_600_ ratio. Three independent assays were performed on different days for each strain, and the data were reported as means ± SD.

### Mouse subcutaneous and intraperitoneal challenge infection models.

Animal studies were reviewed and approved by the Veterans Administration Institutional Animal Care Committee and the University at Buffalo-SUNY and were carried out in strict accordance with the recommendations in the guidelines delineated in the "NIH Guide for the Care and Use of Laboratory Animals" (revised 1985) and the "Ethics of Animal Experimentation Statement" (Canadian Council on Animal Care, July, 1980) as monitored by the Institutional Animal Care and Use Committee (IACUC). All efforts were made to minimize suffering. Veterinary care for the animals was supplied by the staff of the Veterans Administration Animal Facility under the direction of a fully licensed veterinarian. BALB/c, C57BL/6, and CD1 male mice, 4 to 6 weeks old, were obtained from Charles River Laboratories and quarantined for 5 days before use. Bacteria were grown overnight in LB and were subsequently serially diluted to the required titers in 1× phosphate-buffered saline (PBS). A 100-μl bacterial suspension was injected either subcutaneously (SQ) or intraperitoneally (IP) using a 0.5-ml insulin syringe. The bacterial titer was enumerated by serial dilutions of the inoculum. The animals were closely monitored for 14 days after challenge for the development of the study endpoints, survival or severe illness (*in extremis* state)/death, which was recorded as a dichotomous variable. Signs that were monitored and that resulted in immediate euthanasia using methods consistent with the recommendations of the American Veterinary Medical Association Guidelines included hunched posture, ruffled fur, labored breathing, reluctance to move, photophobia, and dehydration. Challenge inocula for all animal experiments are listed in Table S3.

### Statistical analyses.

Statistical analyses were performed using GraphPad Prism 9.0. A log rank (Mantel-Cox) test was performed to analyze *in vivo* infection model data. A Mann-Whitney test was used to compare siderophore concentrations between strain cohorts, and a two-way analysis of variance (ANOVA) was performed for the mucoviscosity assays to compare strain cohorts when grown in LB and c-M9-te medium. Bonferroni posttests were used to account for multiple comparisons. A *P* value of <0.05 was considered statistically significant. *P* values for all animal experiments are listed in Table S4.

10.1128/mSphere.00045-21.4 TABLE S4 *P* values for animal experiments. Download Table S4, XLSX file, 0.01 MB. Copyright © 2021 Russo et al. 2021 Russo et al. https://creativecommons.org/licenses/by/4.0/ This content is distributed under the terms of the Creative Commons Attribution 4.0 International license.
